# Metal–Organic Framework-Mediated Delivery of Nucleic Acid across Intact Plant Cells

**DOI:** 10.1021/acsami.3c19571

**Published:** 2024-04-02

**Authors:** Pei Yu, Xiongjie Zheng, Lukman O. Alimi, Salim Al-Babili, Niveen M. Khashab

**Affiliations:** † Smart Hybrid Materials Laboratory (SHMs), Chemistry Program, Physical Science and Engineering Division, King Abdullah University of Science and Technology (KAUST), Thuwal 23955-6900, Saudi Arabia; ‡ The BioActives Lab, Plant Science Program, Biological and Environmental Science and Engineering Division, King Abdullah University of Science and Technology (KAUST), Thuwal 23955-6900, Saudi Arabia

**Keywords:** metal−organic framework, gene delivery, siRNA, gene silencing, intact plant cells

## Abstract

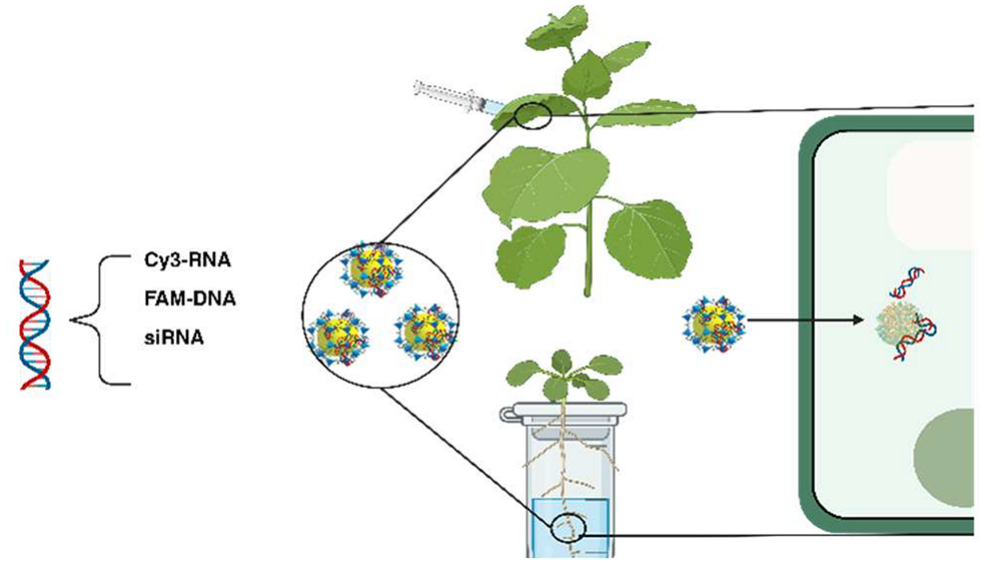

Plant synthetic biology is applied in sustainable agriculture, clean energy, and biopharmaceuticals, addressing crop improvement, pest resistance, and plant-based vaccine production by introducing exogenous genes into plants. This technique faces challenges delivering genes due to plant cell walls and intact cell membranes. Novel approaches are required to address this challenge, such as utilizing nanomaterials known for their efficiency and biocompatibility in gene delivery. This work investigates metal–organic frameworks (MOFs) for gene delivery in intact plant cells by infiltration. Hence, small-sized ZIF-8 nanoparticles (below 20 nm) were synthesized and demonstrated effective DNA/RNA delivery into *Nicotiana benthamiana* leaves and *Arabidopsis thaliana* roots, presenting a promising and simplified method for gene delivery in intact plant cells. We further demonstrate that small-sized ZIF-8 nanoparticles protect RNA from RNase degradation and successfully silence an endogenous gene by delivering siRNA in *N. benthamiana* leaves.

## 1 Introduction

Plant synthetic biology is critical to emerging fields such as sustainable agriculture, algae farming, and clean energy industries. These techniques are mainly employed to improve crop quality,^1^ crop yield,^2^ and pest resistance.^3^ Moreover, plant-based vaccine production has been established for both humans and animals.^4^ In addition, enhancing biofuel production efficiency using genetically modified plants offers a significant advantage for the energy industry.^5^ However, a crucial first step of plant genetic engineering is the introduction of exogenous genes into plant cells and overcoming the hurdle of the inaccessible plant cell wall.

Gene delivery techniques should be deliberated because intact cell membranes impede many exogenous biomolecules, like nucleic acids, from getting into the cytoplasm.^6,7^ There are some conventional methods for delivering genes into plants, including agrobacterium-mediated delivery,^8−10^ biolistic particles,^11^ and electroporation.^12^ Nonetheless, they still suffer from significant drawbacks including being time-consuming and having cell damage, risk of gene damage, and/or expensive production. Therefore, alternative gene delivery strategies are in high demand for delivering exogenous genes into plant cells. Recently, nanomaterials have been utilized for gene delivery for mammal cells because they show excellent features, including notable transformation efficiency, favorable biocompatibility, and effective protection of exogenous nucleic acids.^13−17^

While plant genetic engineering has witnessed significant breakthroughs,^18−20^ it continues to trail behind the advancements in animal genetic engineering. Additionally, the nanomaterial-mediated gene-delivery system in plants is at an early stage, presenting numerous challenges for its widespread application. The plant cell wall imposed a significant hurdle for the development of nanomaterials-mediated gene delivery into plants as it only allows biomolecules with a diameter less than 20 nm to permeate.^21^ Consequently, it would be ideal if the size of rigid nanomaterials were smaller than 20 nm in at least one dimension to facilitate passage through the cell wall. Several researchers successfully delivered exogenous genes into mature plants via nanocarriers. Landry and co-workers have reported gold nanoparticles,^22,23^ carbon nanotubes,^24,25^ and DNA nanostructure^26,27^ based gene delivery systems. Moreover, Schwartz et al. established carbon dots-based RNA delivery.^28^ Li et al. employed functional graphene oxide nanoparticles for gene editing in plants.^29^ These nascent technologies hold the promise of enhancing plant engineering. Moreover, to the best of our knowledge, there is still one kind of favorable nanomaterial, metal–organic frameworks (MOFs), which has not been explored in gene delivery in intact plant cells by simple infiltration.

**Figure 1:**
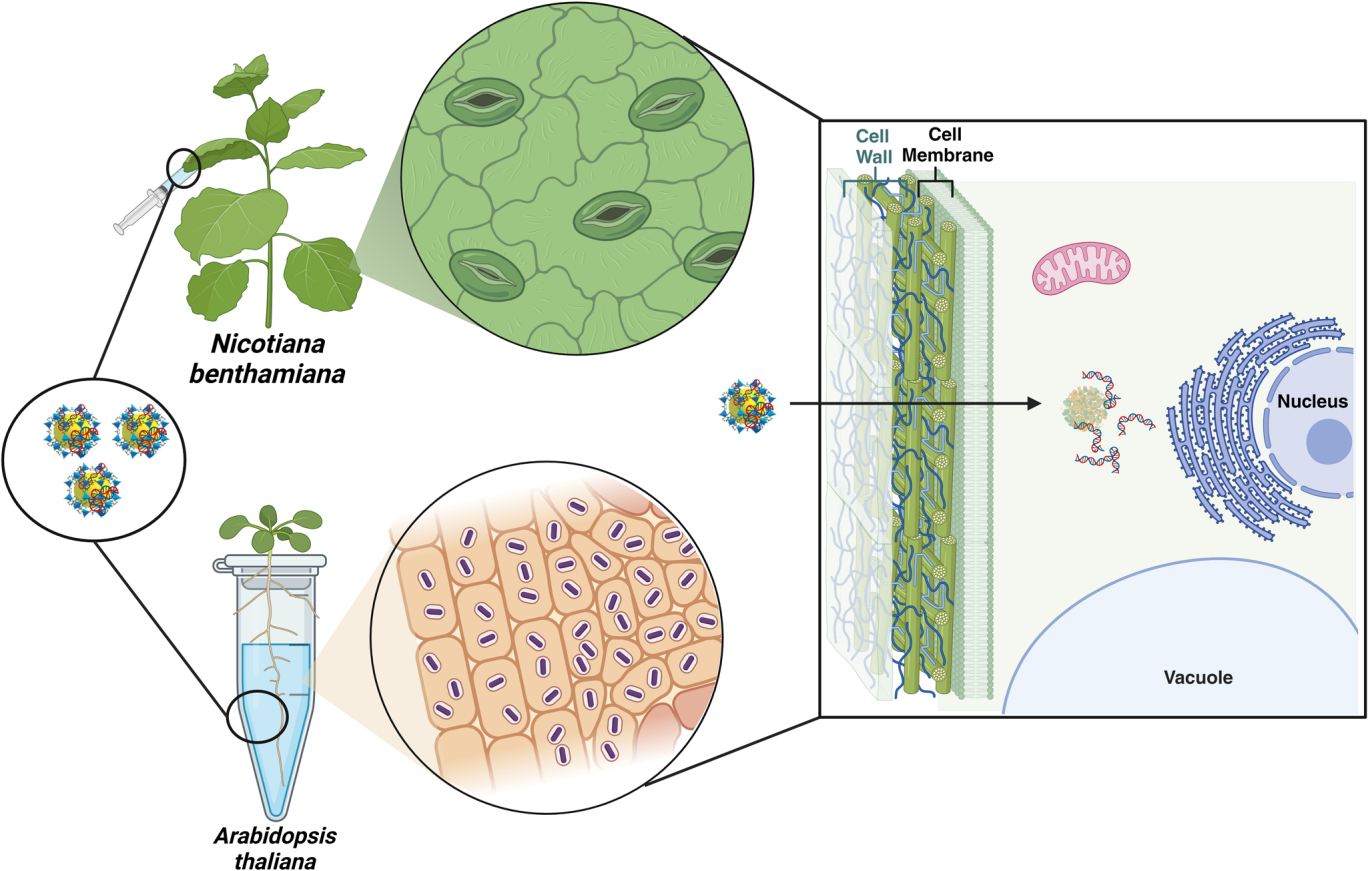
Schematic representation for ZIF-8 NPs mediated gene delivery into *Nicotiana benthamiana* leaves and *Arabidopsis thaliana* root.

Throughout the past decades, many bio-MOFs have been synthesized for biomolecule delivery.^30−35^ Upon these biocompatible nanomaterials, zeolitic imidazolate framework-8 (ZIF-8) nanoparticles are one of the most exploited nanocarriers,^36−39^ which was synthesized through the coordination of Zn^2+^ ions and 2-methylimidazole. Our group previously demonstrated that nanoscaled ZIFs were deployed for controlled codelivery of proteins and sgRNA,^40^ effective and cell-type-specific delivery of CRISPR/Cas9 gene editing elements,^41^ immunotherapeutic delivery,^42^ and auxin delivery in plants.^43^ Herein, small-size ZIF-8 nanoparticles below 20 nm were synthesized for successful delivery of DNA/RNA into *Nicotiana benthamiana* leaves and *Arabidopsis thaliana* root with high efficacy (Figure 1). Furthermore, we show that small-sized ZIF-8 nanoparticles effectively protect RNA from RNase degradation and achieve successful gene silencing by delivering siRNA into *Nicotiana benthamiana* leaves. We proposed a simple, universal, and versatile method for gene delivery in intact plant cells based on biomineralized nanomaterials, which encourages the simplifying of genetic engineering.

## 2 Experimental Section

### 2.1 Chemicals and Materials

All reagents and solvents were purchased from commercial sources and used without further purification. 2-Methyl imidazole, zinc nitrate hexahydrate (Zn(NO_3_)_2_·6H_2_O), and methanol were purchased from Sigma-Aldrich (Darmstadt, German). Phosphate-buffered saline (PBS) buffer and HEPPS buffer were obtained from ThermoFisher (Waltham, MA, USA). DNA was purchased from integrated DNA technologies. RNA and RNase A were purchased from New England BioLabs.

### 2.2 Synthesis of ZIF-8 NPs and Loading of DNA or RNA

ZIF-8 nanoparticles (ZIF-8 NPs) were synthesized according to the previously reported literature with some modifications.^44^ As shown in Figure S1, 172 mg of 2-methyl imidazole was dissolved in 10 mL of methanol and 75 mg of zinc nitrate hexahydrate was separately dissolved in another 10 mL of methanol. To ensure thorough dissolution, a proper ultrasound was required. Next, all the 2-methyl imidazole solution was added dropwise under vigorous stirring at room temperature for 45 min, and white precipitation was obtained after centrifugation. Then, it was filtered through a 10 kDa ultrafiltration membrane (Millipore) to remove extra reagents after dispersion with RNase-free water. After ultrafiltration, DNA or RNA was mixed into the solution to get nucleic acid@ZIF-8 NPs.

### 2.3 ZIF-8 NPs and RNA@ZIF-8 NPs Characterization

The size distribution and surface charge of the prepared nanomaterials were determined using a Zetasizer (NanoZS, Malvern). Transmission electron microscopy (TEM Titan_ST) and scanning electron microscopy (eSEM Quattro) were utilized for sample morphology characterization. Powder X-ray diffraction (PXRD) data were collected by using an X-ray diffractometer (D2 PHASER XE-T, Bruker).

### 2.4 UV–vis Pattern

Pure RNA, ZIF-8 NPs, and RNA@ZIF-8 NPs were dispersed in water and transferred into a quartz cuvette with a 10 mm path length. Absorption spectra were recorded on a Thermo Evolution 600 UV–visible spectrophotometer.

### 2.5 ZIF-8 NPs Degraded in PBS

ZIF-8 NPs were diffused into the water and PBS (pH 5.5) solution, respectively, then incubated at 37 °C several times. After that, the solution was dropped on the grid and tested with transmission electron microscopy (TEM Titan_ST) after drying. Scanning electron microscopy (eSEM Quattro) and PXRD (D2 PHASER XE-T, Bruker) were utilized to analyze the degradation of ZIF-8 NPs.

### 2.6 RNA Loading Capacity Measurements and Release Gel Assay

To determine the loading capacity of ZIF-8 NPs, 100 ng RNA was mixed with ZIF-8 NPs at various mass ratios (1:15, 1:30, 1:45, 1:60, 1:75, 1:90, 1:105) in DI water. The prepared samples were characterized by 1% agarose gel electrophoresis. To detect the release of RNA, 100 ng RNA was mixed with 0.75 μg ZIF-8 NPs, and the mixture was diluted in water or an acidic solution (pH 3) and incubated for 5 min. The prepared samples were characterized by 1% agarose gel electrophoresis.

### 2.7 RNA Protection Gel Assay

For RNA protection assay, 100 ng free RNA was added into DI water or ZIF-8 NPs solution (1:75). Next, the RNase A (10 μg/mL) was added into the mixtures and incubated for 0, 5, 10, 20, and 30 min. The samples were heated to 95 °C for 5 min to deactivate the enzyme after incubation. Then, the prepared samples were characterized by 1% agarose gel electrophoresis.

### 2.8 Plant Growth and Maintenance

Wild-type *N. benthamiana* (*Nb*) seeds were grown in individual 100 mm pots under LED light in the artificial greenhouse where the environment had a 14/10 light/dark photoperiod at 23 °C and 60% humidity. All experiments in this study were performed on the healthy and intact leaves of 5–6 weeks plants.

### 2.9 Leaf Infiltration of ZIF-8 NPs

A small puncture hole was created on the abaxial surface of the *Nb* plant leaf using a 10 μL pipet tip before infiltrating the leaves. The infiltration process involved gently pushing approximately 100 μL of fluid into the leaf tissue using a needle-less syringe with a 1 mL capacity.

### 2.10 Internalization of Cy3-RNA@ZIF-8 NPs into GFP Expressed *Nb* Plant Leaf Cells Quantified through Colocalization Analysis

The complete CDS of green fluorescent protein (GFP) was cloned into the pDONR221 vector and was then recombined into the plant overexpression gateway vector pB2GW7 using an LR reaction kit (Invitrogen) to generate pB2GW7-GFP. The pB2GW7-GFP plasmid was transformed into GV3101 *Agrobacterium tumefaciens* using electroporation. The *Agrobacterium*-mediated transient expression in 5-week-old *N. benthamiana* leaves was performed as previously described. The OD600 of pB2GW7-GFP *Agrobacterium* was adjusted to 0.6 before infiltration. To investigate the delivery of double-stranded RNA (dsRNA) by ZIF-8 NPs, *Nb* plants were cultivated for 2 days after infiltrating pB2GW7-GFP *Agrobacterium* to ensure the expression of GFP in *Nb* leaf cells.

Then, pure Cy3-RNA, pure ZIF-8 NPs, and Cy3-RNA@ZIF-8 NPs were dispersed into water as the RNA final concentration is 3 μg/mL. Then, three of them were infiltrated in the GFP-overexpressing leaves but a different area separately and kept in the plant growth room for 4 h incubation. A small section of the infiltrated leaf was cut and positioned between a glass slide and a coverslip of a specific thickness. Water was applied to maintain hydration of the leaf sections during the imaging process. Plant tissue was imaged using a Leica SP8 confocal laser scanning microscope, employing 488 and 543 nm laser excitation for the collection of GFP and Cy3 signals, respectively. The images were captured at 20× magnification.

### 2.11 Quantitative Real-time PCR (qRT-PCR) Experiments for Gene Silencing Confirmation

The gene silencing was assessed by targeting a gene responsible for encoding a H subunit of magnesium chelatase (*cHLH*), a crucial enzyme involved in chlorophyll synthesis in *N. benthamiana*.^28^ Water (control), pure *cHLH*, *cHLH*@ZIF-8 NPs, and nonfunctional siRNA@ZIF-8 NPs were infiltrated into plant leaves and remained 1 day before RNA extraction. Total RNA of infiltrated leaf samples was extracted using Trizol reagent and Direct-zol RNA Miniprep Plus Kit (Zymo, Irvine, CA, USA). The cDNA was then synthesized by using iScript cDNA Synthesis Kit (BIO-RAD, USA) following the manufacturer’s protocol. qRT-PCR was conducted on an Applied Biosystems StepOnePlus Real-Time PCR System following the manufacturer’s instruction of SsoAdvanced Universal SYBR Green Supermix kit (BIO-RAD, USA). *N. benthamiana* Actin (AY179605) was used as a reference gene to normalize the expression of the *cHLH* gene. The E^*–*ΔΔ*Ct*^ method was used to calculate the relative expression of *cHLH*. The qRT-PCR primers are shown in the Supporting Information.

### 2.12 DNA Delivery into *Nb* Plant Leaf Cells

Dispersed in water with a single-stranded DNA (ssDNA) concentration of 3 μg/mL, pure FAM-DNA, pure ZIF-8 NPs, and FAM-DNA@ZIF-8 NPs were separately infiltrated into distinct areas of the same leaves, undergoing a 4 h incubation in the plant growth room. Following this, small leaf sections at the infiltration sites were cut and positioned between glass slides and coverslips, with water applied for hydration during imaging. Utilizing a Leica SP8 confocal microscope at 488 nm, plant tissue was imaged at 20× magnification.

### 2.13 DNA Delivery into *Arabidopsis thaliana* Root Cells

Pure FAM-DNA, ZIF-8 NPs, and FAM-DNA@ZIF-8 NPs (final DNA concentration: 3 μg/mL) were dispersed in water and applied to *Arabidopsis thaliana* roots, which were immersed in centrifuge tubes for 2 days with water added every 12 h. Then, part of the root was cut and washed with 1 × PBS for several times. The sections, placed between a glass slide and a coverslip of specific thickness, were hydrated with water to maintain moisture during the imaging process. Samples were imaged as *Nb* plant leaf cells.

## 3 Results and Discussion

### 3.1 Synthesis and Characterization of ZIF-8 Nanoparticles

As illustrated in Figure S1, ZIF-8 nanomaterials were prepared by adding 2-methylimidazole (C_4_H_6_N_2_) solution into zinc nitrate hexahydrate in methanol.^44^ Turbid mixture observed after 45 min by nanoparticle precipitation. Then, the sediment was washed with water and centrifuged to remove extra reagents. As shown in Figure S2, the transmission electron microscopy (TEM) image showed that these nanoparticles displayed a spherical morphology with mean sizes of 16 ± 4 nm. The histograms of ZIF-8 NPs’ size distribution also demonstrate that most ZIF-8 NPs’ sizes are below 20 nm (Figure S3). The powder X-ray diffraction (PXRD) patterns of the synthesized ZIF-8 nanoparticles are identical to the simulated ZIF-8 (Figure S5). These results imply that small-size ZIF-8 nanoparticles were synthesized, which is more possible to travel across plant cell walls (pore size below 20 nm). Degradation of ZIF-8 nanoparticles in weak acidic PBS was also investigated because the pH of plant cells’ interspace and vacuole is about 5.5.^45,46^ The TEM, SEM, and PXRD results show ZIF-8 NPs were degraded after being incubated in pH 5.5 PBS for 10 min (Figures S5–S7).

### 3.2 RNA Loaded on ZIF-8 Nanoparticles

**Figure 2:**
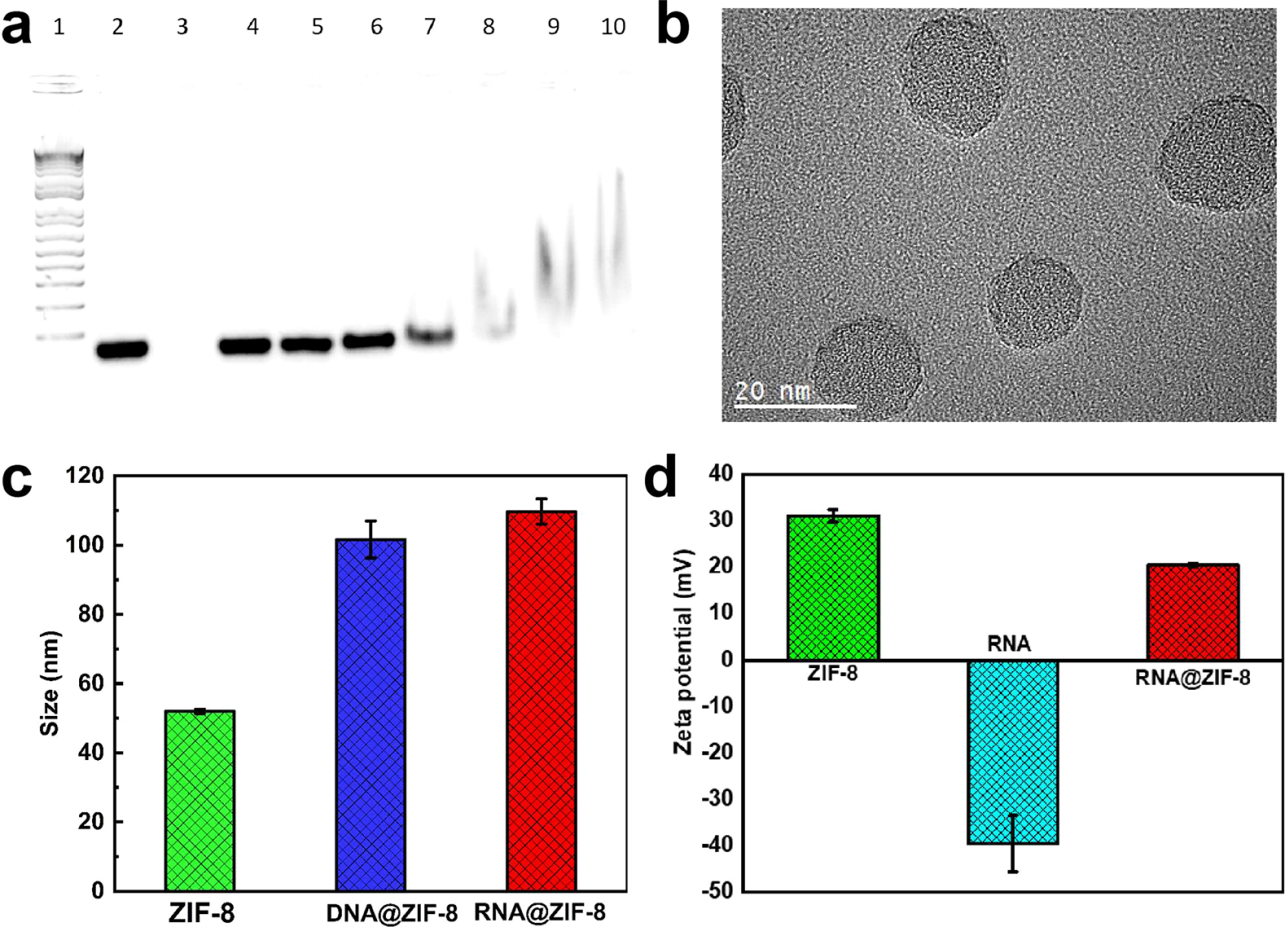
RNA loading onto ZIF-8 nanoparticles. (a) Agarose gel electrophoresis of 100 ng RNA loaded on ZIF-8 NPs at different mass ratios. Lane 1: marker; lane 2: pure RNA; lane 3: pure ZIF-8 NPs; lanes 4 to 10 (RNA/ZIF-8 NPs): 1/15; 1/30; 1/45; 1/60; 1/75; 1/90; and 1/105 (w/w). (b) Transmission electron microscopy (TEM) image of RNA@ZIF-8 NPs on the 20 nm scale. (c) Dynamic light scattering (DLS) measurement of ZIF-8 NPs (green), DNA@ZIF-8 NPs (blue), and RNA@ZIF-8 NPs (red). (d) Zeta potential of ZIF-8 NPs (green), pure RNA (cyan), and RNA@ZIF-8 NPs (red).

RNA and ZIF-8 nanoparticles were mixed at different mass ratios to assess RNA loading efficiency (RNA/ZIF-8 NPs: 1/5 to 1/105). As shown in Figure 2a, RNA was completely loaded at the ratios of RNA:ZIF-8 NPs = 1:75, where we utilized the ratio for later measurement. No noticeable morphology change was observed after loading RNA into ZIF-8 NPs from TEM images (Figure 2b and Figure S2). Dynamic light scattering (DLS) measurements gave the average diameters of 51.9 and 101.6 nm for ZIF-8 and RNA@ZIF-8 NPs, respectively (Figure 2c and Figure S4). The increase in the DLS diameter for RNA@ZIF-8 NPs was consistent with the presence of RNA probes on the ZIF-8 surface. The zeta potential result is provided (Figure 2d), the surface charge changed from 31.1 mV to 20.5 mV after loading RNA, and the DLS and zeta potential results were summarized in Table S2–3. The UV–vis (Figure S8) pattern showed RNA loaded. To investigate RNA release efficiency, we observed a clear RNA band after dissolving ZIF-8 in acidic hepps buffer (Figure S9).

### 3.3 RNA Delivery into *Nicotiana benthamiana* Leaves

**Figure 3:**
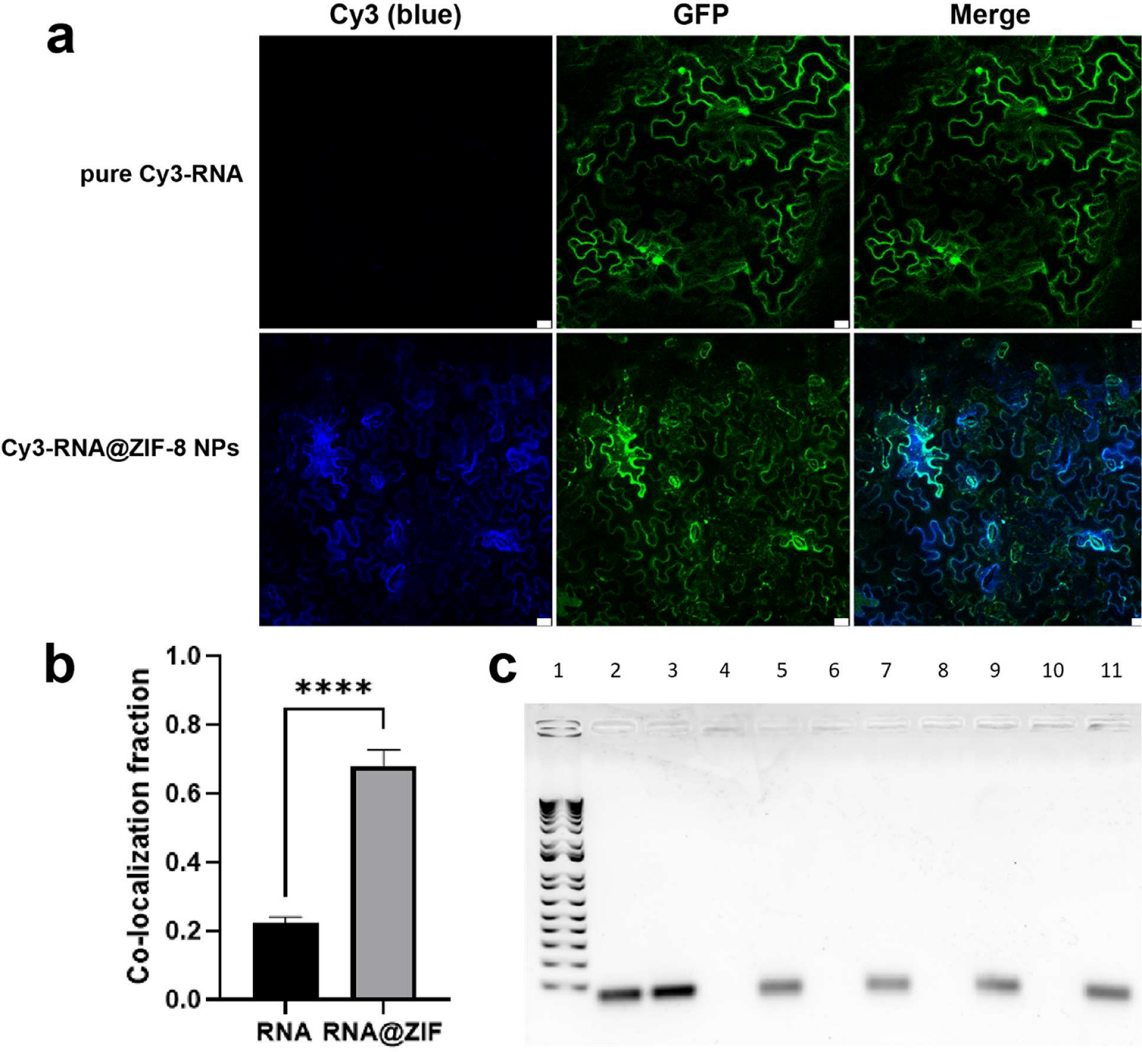
Internalization and nuclease protection of Cy3-labeled RNA loaded ZIF-8 nanoparticles. (a) Representative confocal images showing the internalization of pure Cy3-labeled RNA and Cy3-labeled RNA-loaded ZIF-8 NPs into cells of GFP overexpressing *N. benthamiana* leaves, scale bar: 20 μm. (b) Quantitative colocation fluorescence intensity analysis of transgenic *Nb* treated with pure Cy3-labeled RNA and Cy3-labeled RNA@ZIF-8 NPs. (c) Agarose gel electrophoresis with RNA alone or RNA@ZIF-8 NPs incubated with RNase for different time. Lane 1: marker; lanes 2–3: 0 min; lanes 4–5: 5 min; lanes 6–7: 10 min; lanes 8–9: 20 min; and lanes 10–11: 30 min.

Next, we assessed the ability of ZIF-8 NPs to mediate RNA delivery into the cytosol of mature *N. benthamiana* leaf cells. First, the plants were infiltrated with *A. tumefaciens* GV3101 containing the GFP overexpressing vector to provide an intracellular fluorescent marker. As a result, green fluorescence appeared in the cytosol and nucleus of plant cells after 2 days infiltration (Figure S10). Next, RNA@ZIF-8 nanoparticles were dispersed in water at an RNA concentration of 3 μg/mL, 100 μL of apparent solution were infiltrated to *N. benthamiana* leaves. The same concentration of pure RNA also was tested as a control, and the tissues were collected after 4 h of infiltration. We observed enhanced Cy3 fluorescence in the nanoparticle group compared with pure RNA (Figure 3a). The colocalization between Cy3 fluorescence intensity and GFP quantification of CLSM images are shown in Figure 3b, which increased over 50%. Furthermore, there is no obvious damage on the leaves after infiltration for 3 days (Figure S13). Previous reports have shown that fragile RNA were easily degraded by RNase in the environment. To investigate the protection of RNA by ZIF-8 NPs against RNase conditions, 10 μg RNase A was incubated with RNA@ZIF-8 NP where the RNA amount is 100 ng for different periods of time. Gel electrophoresis results indicated that the RNA loaded onto ZIF-8 NPs was greatly protected from degradation, while free RNA was completely digested, as seen from the absence of an RNA band (Figure 3c).

### 3.4 Functional RNA Delivery into *Nicotiana benthamiana* Leaves

In addition, we chose a 22-bp siRNA sequence that is able to silence the *cHLH* gene in the *N. benthamiana* plant (ref) to evaluate the ability of ZIF-8 NPs to serve as a siRNA delivery tool for transient gene silencing. To do this, we infiltrated the functional-siRNA@ZIF-8 NPs, 3 μg/mL siRNA loaded on the surface of ZIF-8 NPs, pure water, siRNA, and nonfunctional RNA@ZIF-8 NPs into *N. benthamiana* leaves, respectively. The infiltrated leaf tissues were collected for total RNA extraction. The qRT-qPCR analysis was employed to quantify relevant mRNA level in the cells. As shown in Figure S11, compared with other controls, the functional siRNA with ZIF-8 NPs resulted in a significant decrease in mRNA level in infiltrated leaves, indicating that ZIF-8 NPs achieve functional siRNA delivery.

### 3.5 DNA Delivery into Nicotiana Benthamiana Leaves and *Arabidopsis thaliana* Root

**Figure 4:**
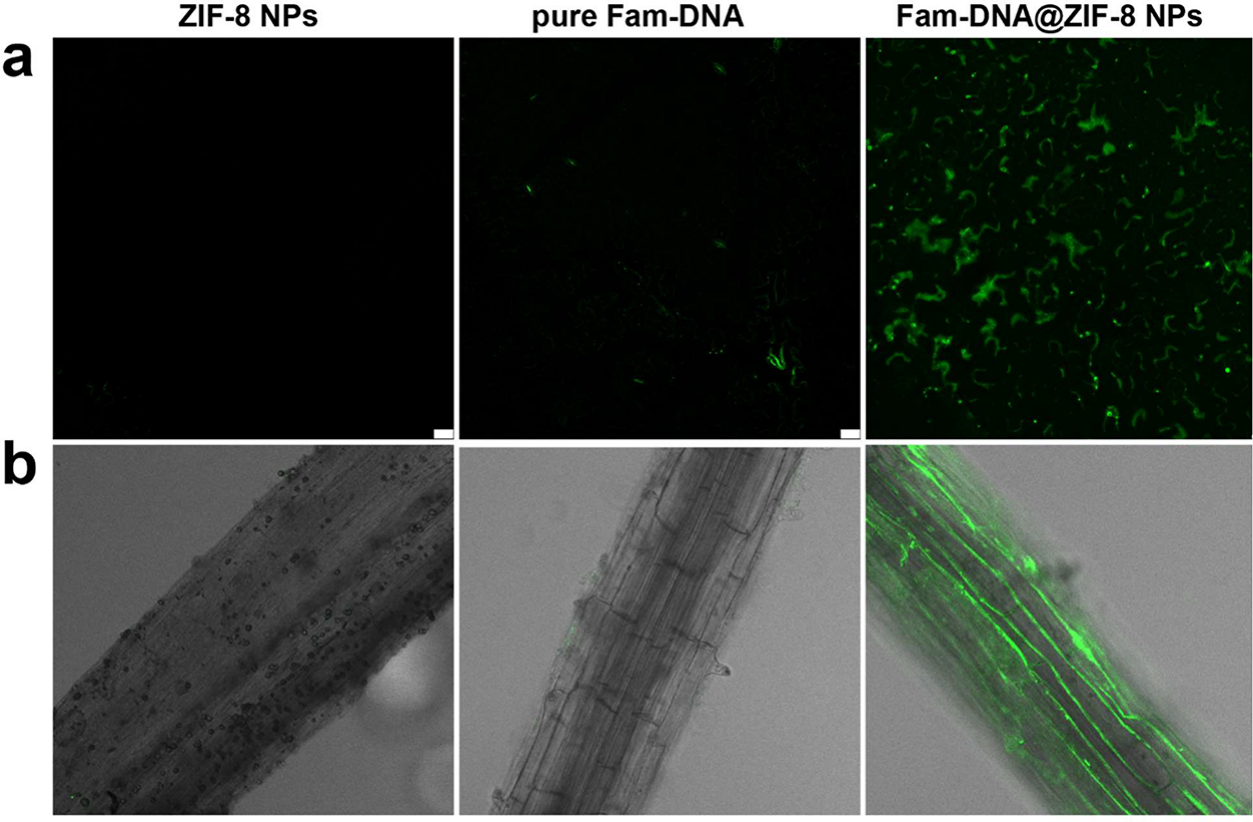
Confocal images of *Nicotiana benthamiana* leaves and *Arabidopsis thaliana* root after infiltration. (a, b) Representative confocal images showing pure ZIF-8 NPs, pure Fam-labeled DAN, and Fam-labeled DNA-loaded ZIF-8 NPs into cells of *Nicotiana benthamiana* leaves and *Arabidopsis thaliana* root by infiltration, scale bar: 20 μm.

Next, we tested DNA delivery in leaves and roots. As we treated with RNA delivery, the same concentration of FAM-labeled DNA loaded ZIF-8 NPs and pure FAM-DNA were diluted into water and then infiltrated into plant leaves. Apparent FAM fluorescence was detected after treatment, while there was only a weak signal in the pure FAM-DNA group, which means more DNA was sent into cells by our nanoparticles (Figure 4a and Figure S12). Moreover, we collected noninfiltrated areas in the same leaves; no fluorescence was detected, suggesting our system did not affect the untreatment region (Figure S14). The *Arabidopsis thaliana* roots were also employed to evaluate DNA delivery into root cells. The plants were immersed in water containing pure FAM-DNA, ZIF-8 NPs, and FAM-DNA@ZIF-8 NPs for 24 h (Figure S15). Then, the roots were collected for confocal images after washing with PBS for three times. The results showed DNA delivered with the assistance of ZIF-8 NPs (Figure 4b, Figures S16–S17).

## 4 Conclusion

In this work, the small size of ZIF-8 nanoparticles was constructed by modifying the synthesis procedures from literature. And then DNA or RNA were loaded on the surface of nanoparticles by electrostatic attraction. Finally, we successfully delivered DNA and RNA into *Nicotiana benthamiana* leaves and *Arabidopsis thaliana* root by infiltration. Therefore, small size ZIF-8 nanoparticles could be a promising delivery platform for delivery DNA and RNA to accomplish some applications, such as gene editing, cell imaging, and biosensing.

## Supporting Information Available

TEM and SEM imaging, PXRD and UV–vis spectra, in addition to quantitative release assays (PDF)
