# Supplementary material for: Metal–Organic Framework-Mediated Delivery of Nucleic Acid across Intact Plant Cells
Source: ACS Appl Mater Interfaces. 2024 Apr 2;16(15):18245–51. doi: 10.1021/acsami.3c19571 (PMC11040530; doi:10.1021/acsami.3c19571)
Supplement: Supplementary file 1 — am3c19571_si_001.pdf [file am3c19571_si_001.pdf]

Supporting Information

Metal-Organic Framework-Mediated Delivery of Nucleic Acid across Intact Plant Cells

Pei Yu,‡1 Xiongjie Zheng,‡2 Lukman O. Alimi,1 Salim Al-Babili,2 Niveen M. Khashab1**

1. *Smart Hybrid Materials Laboratory (SHMs), Chemistry Program, Physical Science and Engineering Division, King Abdullah University of Science and Technology (KAUST), Thuwal 23955-6900, Saudi Arabia*

2. *The BioActives Lab. Plant Science Program, Biological and Environmental Science and Engineering Division, King Abdullah University of Science and Technology (KAUST), Thuwal 23955-6900, Saudi Arabia*

*Corresponding author:

Niveen M. Khashab

niveen.khashab@kaust.edu.sa

Salim Al-Babili

salim.babili@kaust.edu.sa

‡ Pei Yu and Xiongjie Zheng contributed equally to this work

Supporting Information:

Table S1. Sequences of oligonucleotides used in this study.

Table S2. Zeta potential of ZIF-8 NPs, RNA and RNA@ZIF-8 NPs.

Table S3. The DLS data of DNA@ZIF-8 NPs, RNA@ZIF-8 NPs and ZIF-8 NPs.

Figure S1. Scheme of ZIR-8 NPs, gene@ZIF-8 NPs synthesis.

Figure S2. TEM image of ZIF-8 NPs, DNA@ZIF-8 NPs and RNA@ZIF-8 NPs.

Figure S3. Histogram of ZIF-8 NPs size distribution according to TEM images.

Figure S4. DLS results of ZIF-8 NPs, DNA@ZIF-8 NPs and RNA@ZIF-8 NPs.

Figure S5. PXRD patterns of ZIF-8 NPs, simulated ZIF-8 and ZIF-8 NPs after incubation in pH = 5.5 PBS buffer.

Figure S6. TEM image of (a) ZIF-8 NPs in water, (b) ZIF-8 NPs in pH equal to 5.5 PBS buffer.

Figure S7. SEM image of (a) ZIF-8 NPs in water, (b) ZIF-8 NPs in pH equal to 5.5 PBS buffer.

Figure S8. UV-Vis spectra of pure RNA, pure ZIF-8 NPs and RNA@ZIF-8 NPs.

Figure S9. Release of RNA after dissolved ZIF-8 NPs in Hepps buffer with 1% of agarose gel.

Figure S10. Subcellular areas in GFP *Nicotiana benthamiana* leaf cells.

Figure S11. *cHLH* mRNA fold changes 1day post-infiltration with water(control), pure functional-siRNA, non-functional RNA@ZIF-8 NPs and functional RNA@ZIF-8 NPs.

Figure S12. Quantitative fluorescence intensity analysis of confocal images for DNA, DNA@ZIF-8 NPs and ZIF-8 NPs infiltrated *Nicotiana benthamiana* leaf cells.

Figure S13. The tissue influence caused by infiltration. Pure DNA and DNA@ZIF-8 NPs solution treated leaf after 1 and 3 days.

Figure S14. Representative CLSM images of no-treatment area in the same leaf.

Figure S15. Photographs of *Arabidopsis thaliana* root immersed into mixed pure FAM-DNA or FAM-DNA@ZIF-8 NPs solution.

Figure S16. Representative CLSM images of *Arabidopsis thaliana* root cells treated with ZIF-8 NPs, pure FAM-DNA and FAM-DNA@ZIF-8 NPs.

Figure S17. Quantitative analysis of fluorescence intensity in confocal images was conducted for DNA, DNA@ZIF-8 NPs, and ZIF-8 NPs infiltrated into *Arabidopsis thaliana* root cells.

Table S1: Sequences of oligonucleotides used in this study.

Name	Sequence
Cy3-RNA	3'-AUGUUGUCGGUGUUGCAGAUA-5'<br>5'-UACAACAGCCACAACGUCUAU-Cy3-3'
FAM-DNA	5'-FAM-CCGCGGCCAGGCTACCTACAACGACCTGGACGA-3'
Functional siRNA (*cHLH*)	3'-GAAAACGGAGGUUAAGGUACUA-5'<br>5'-UUUGCCUCCAAUUCCAUGAUCA-3'
Non-functional RNA	3'-AUGUUGUCGGUGUUGCAGAUA-5'<br>5'-UACAACAGCCACAACGUCUAU-3'
cHLH-RT-F	5'-GCTAATGCTCAGGTACGAACG-3'
cHLH-RT-R	5'-CTCAATCTCACGAACTCCCTC-3'
Actin-RT-F	5'-TCACAGAAGCTCCTCTTAATCC-3'
Actin-RT-R	5'-GGGAAAGAACAGCCTGAATG-3'

Table S2: Zeta potential of ZIF-8 NPs, RNA and RNA@ZIF-8 NPs.

Name	Zeta potential (mV)
ZIF-8 NPs	31.1 ± 1.344
Non-functional RNA	-39.47 ± 6.058
Non-functional RNA@ZIF-8 NPs	20.47 ± 0.309

Table S3: The DLS data of DNA@ZIF-8 NPs, RNA@ZIF-8 NPs and ZIF-8 NPs.

Name	DLS (nm)
ZIF-8 NPs	51.93 ± 0.62
FAM-DNA@ZIF-8 NPs	101.62 ± 5.34
Non-functional RNA@ZIF-8 NPs	109.7 ± 3.63

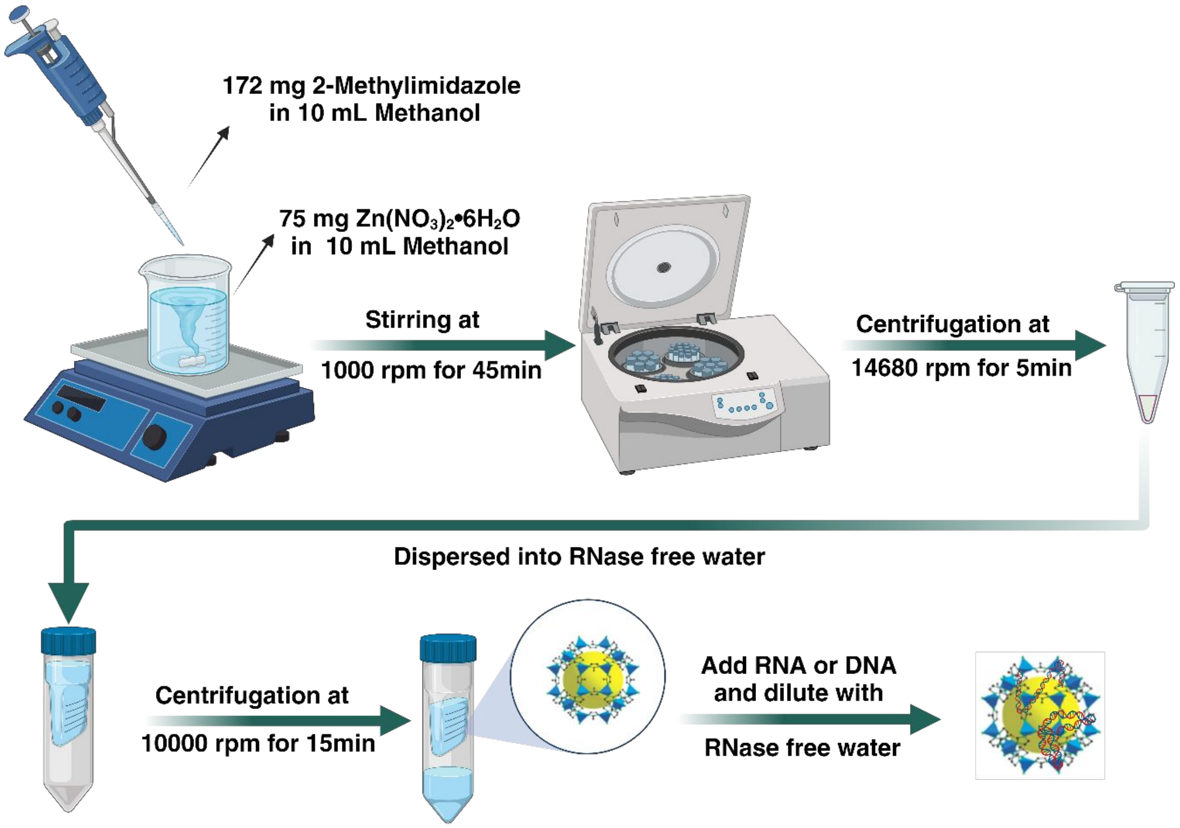

Figure S1. Scheme of ZIR-8 NPs, gene@ZIF-8 NPs synthesis.

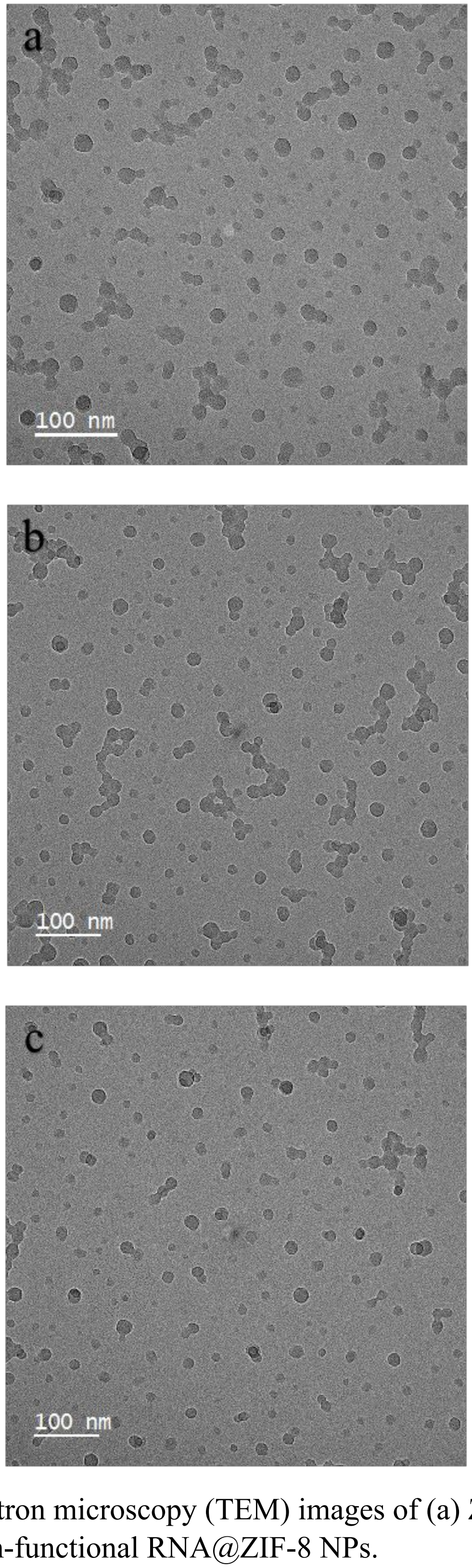

Figure S2. Transmission electron microscopy (TEM) images of (a) ZIF-8 NPs, (b) FAM-DNA@ZIF-8 NPs and (c) non-functional RNA@ZIF-8 NPs.

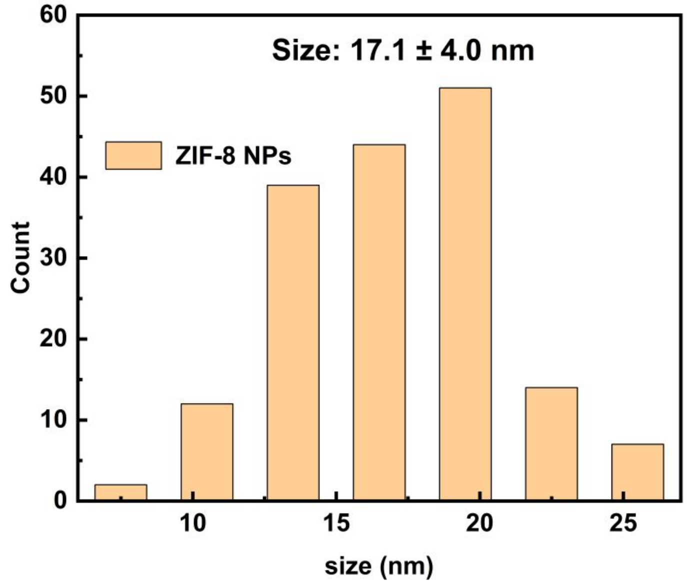

Figure S3. Histogram of ZIF-8 NPs size distribution according to TEM images.

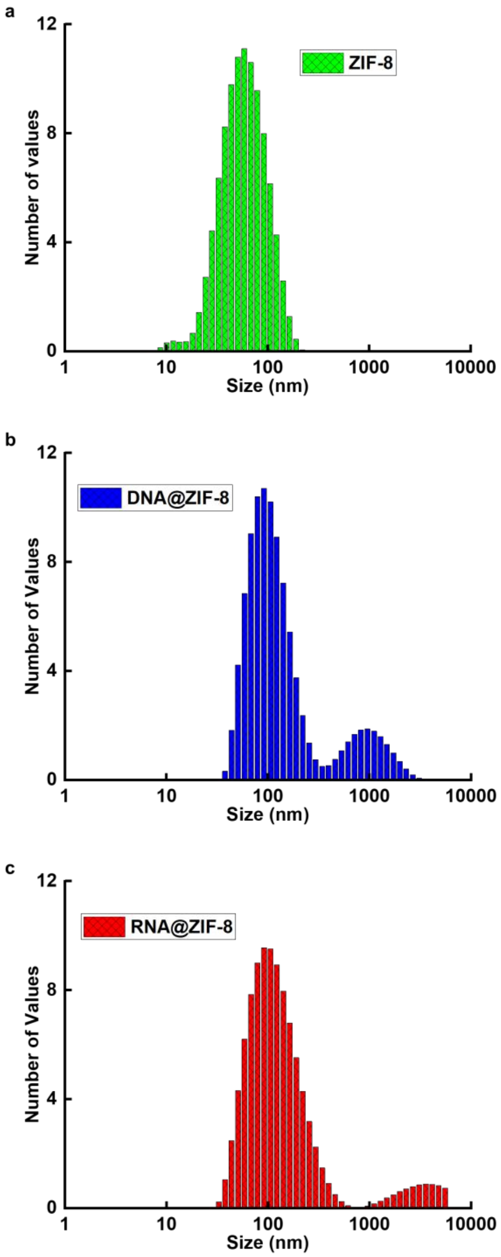

Figure S4. DLS results of ZIF-8 NPs, FAM-DNA@ZIF-8 NPs and non-functional RNA@ZIF-8 NPs.

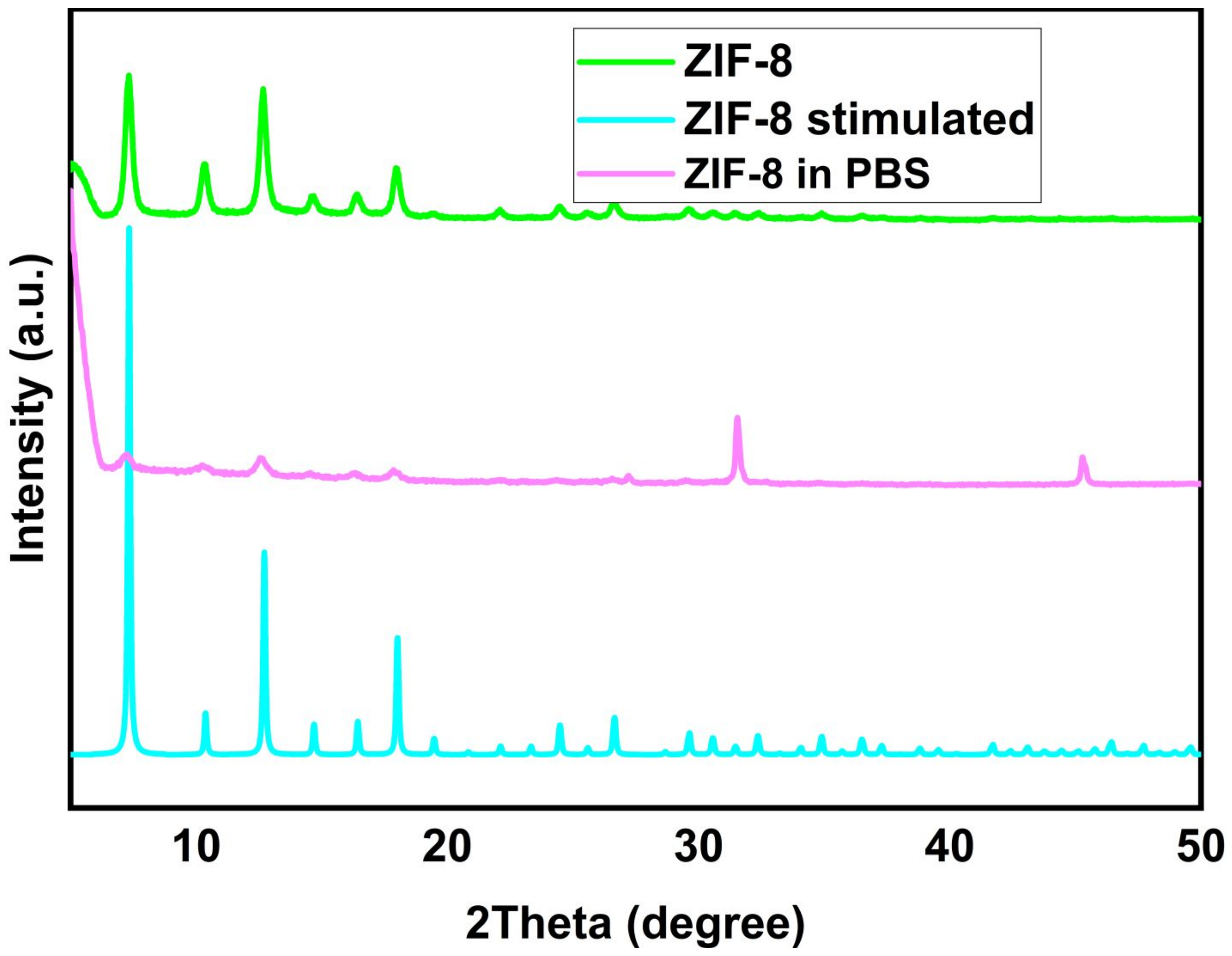

Figure S5. PXRD patterns of ZIF-8 NPs, simulated ZIF-8 and ZIF-8 NPs after incubation in pH = 5.5 PBS buffer.

.

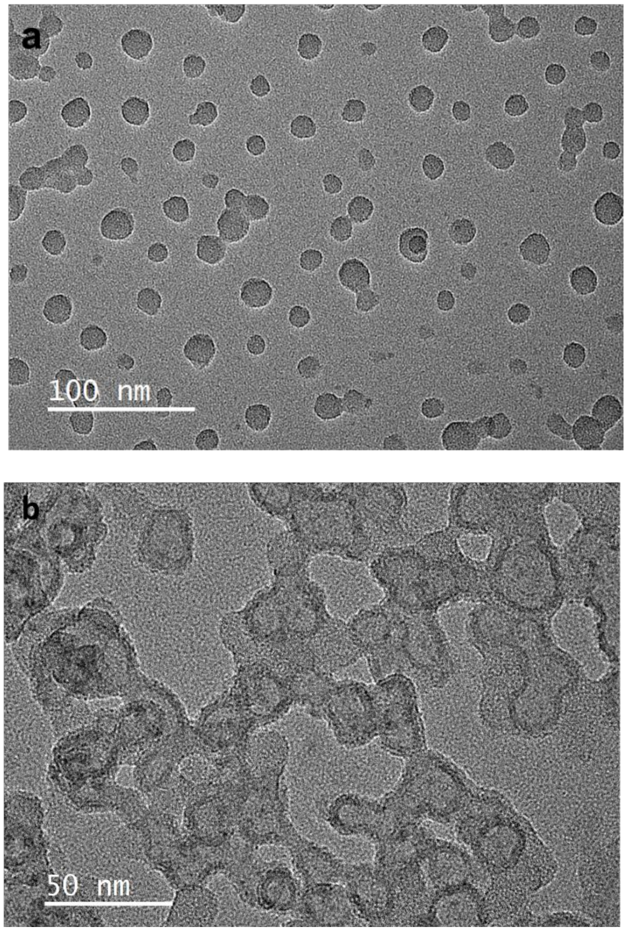

Figure S6. TEM image of (a) ZIF-8 NPs in water, (b) ZIF-8 NPs dispersed in pH equal to 5.5 PBS buffer for 10 mins.

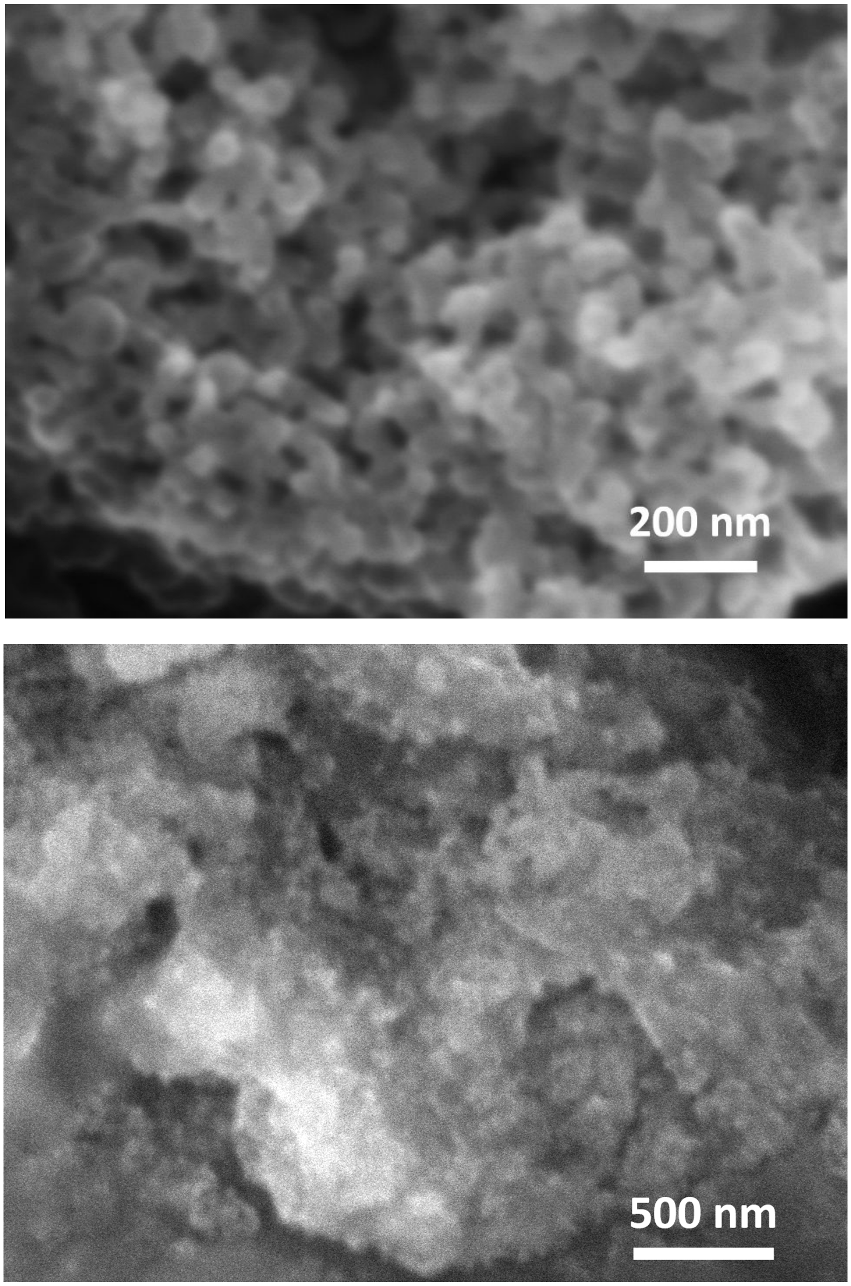

Figure S7. SEM image of ZIF-8 NPs in water (up) and ZIF-8 NPs in pH equal to 5.5 PBS buffer for 10 mins (down).

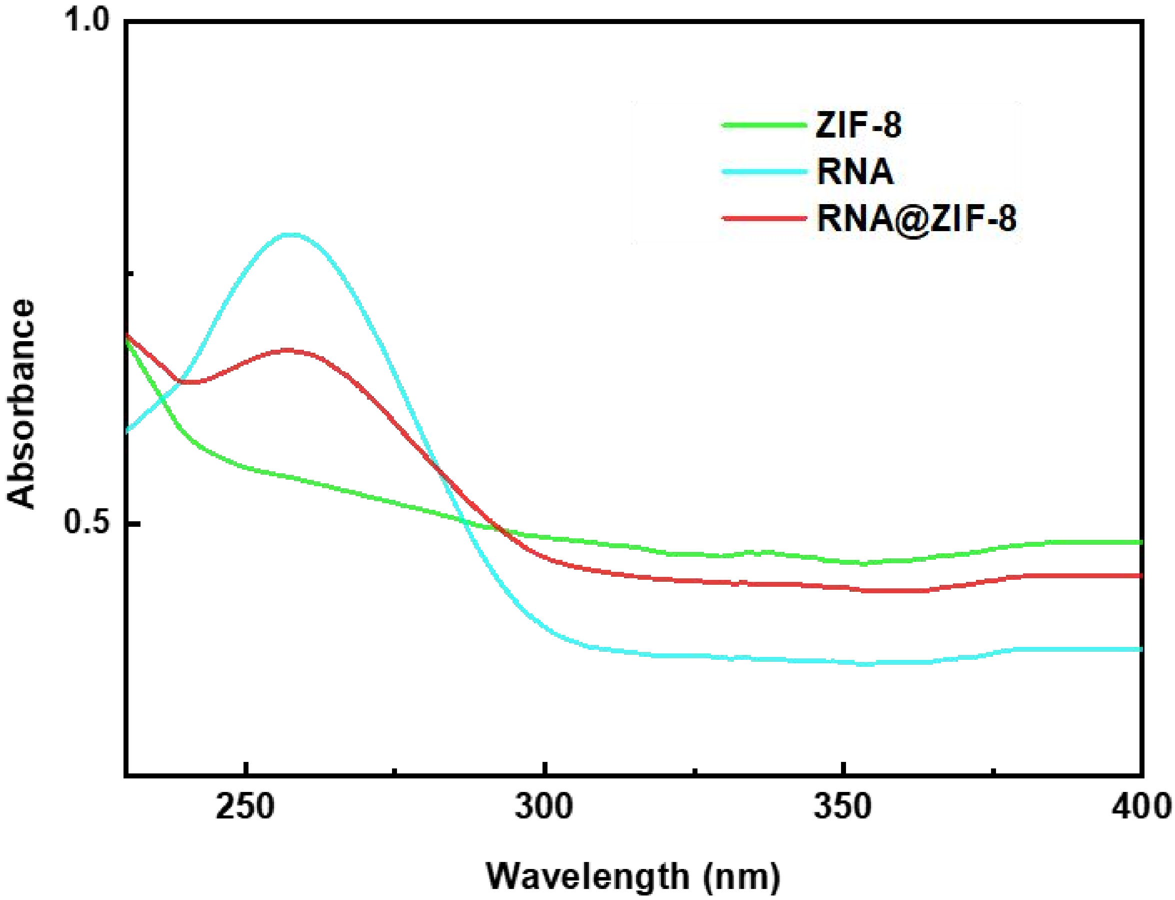

Figure S8. UV-Vis spectra of pure non-functional RNA, pure ZIF-8 NPs and non-functional RNA@ZIF-8 NPs.

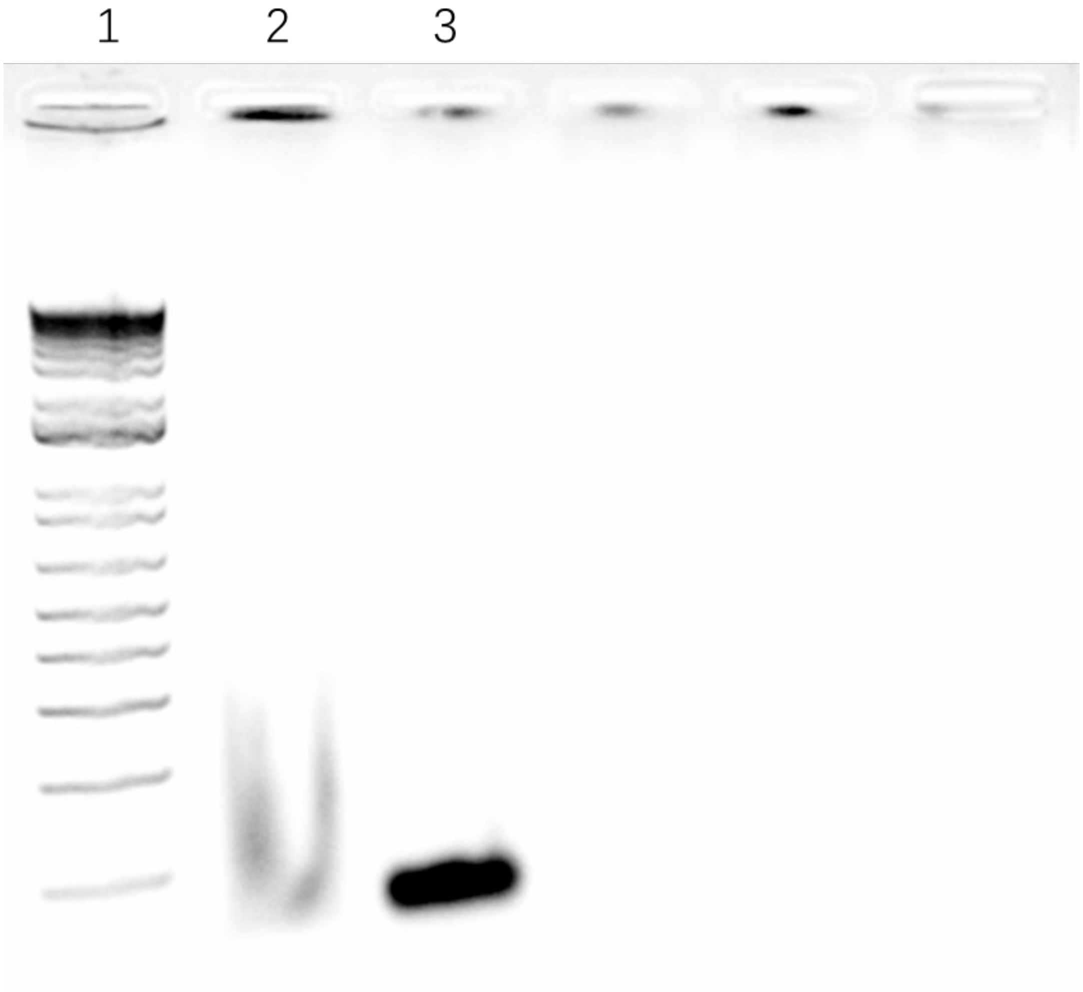

Figure S9. Release of RNA after dissolve ZIF-8 NPs in Hepps buffer with 1% of agarose gel. Lane 1: marker; lane 2: non-functional RNA@ZIF-8 NPs; lane 3: non-functional RNA@ZIF-8 NPs dissolved in HCl solution (pH 3).

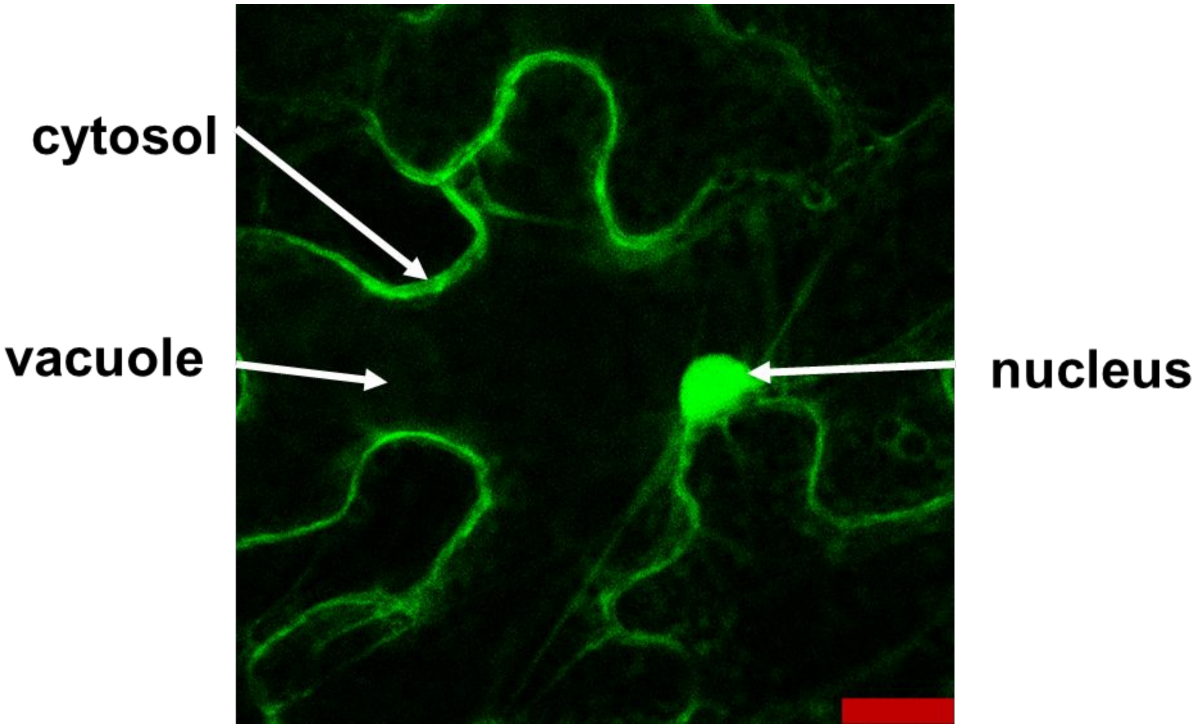

Figure S10. Subcellular areas in GFP *Nicotiana benthamiana* leaf cells. GFP expressed on cytosol and nucleus. Scale bar: 20 µm.

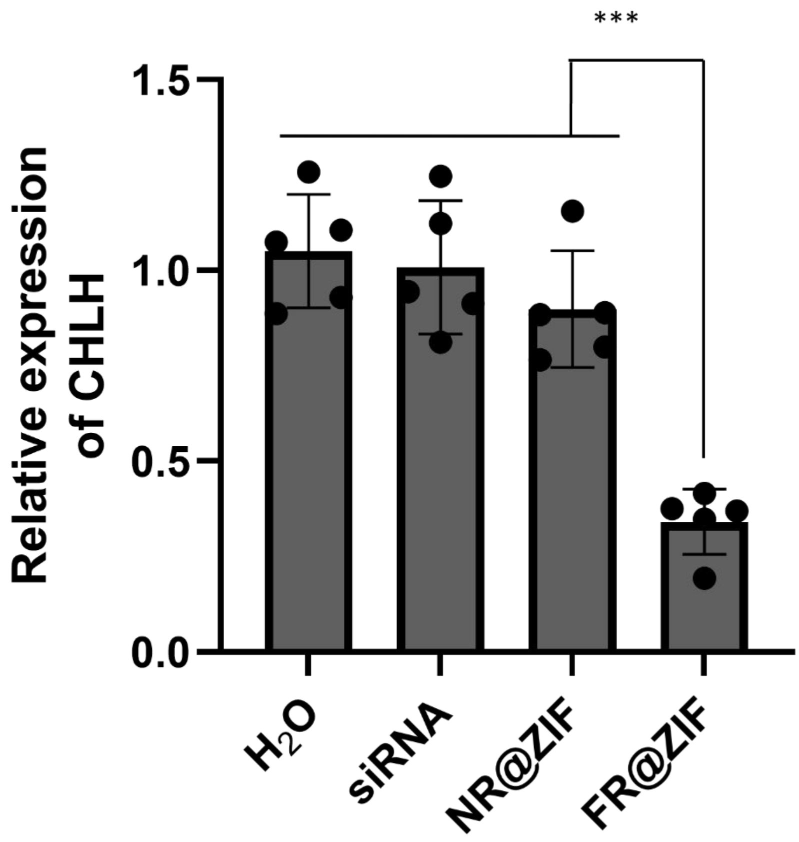

Figure S11. siRNA delivered by ZIF-8 NPs can induce gene silencing. qPCR to quantify *cHLH* mRNA fold changes 1day post-infiltration with water(control), pure functional-siRNA, non-functional RNA@ZIF-8 NPs (NR@ZIF-8) and functional RNA@ZIF-8 NPs (FR@ZIF). ($n = 5$, and ***$P < 0.001$).

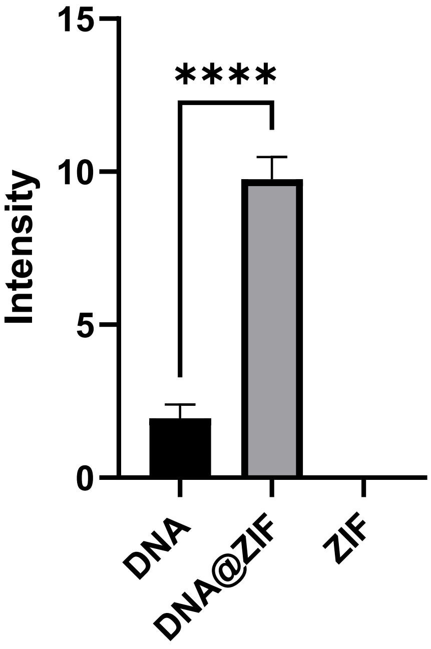

Figure S12. Quantitative fluorescence intensity analysis of confocal images for DNA, DNA@ZIF-8 NPs and ZIF-8 NPs infiltrated *Nicotiana benthamiana* leaf cells. ****$P < 0.0001$, SD ($n = 7$).

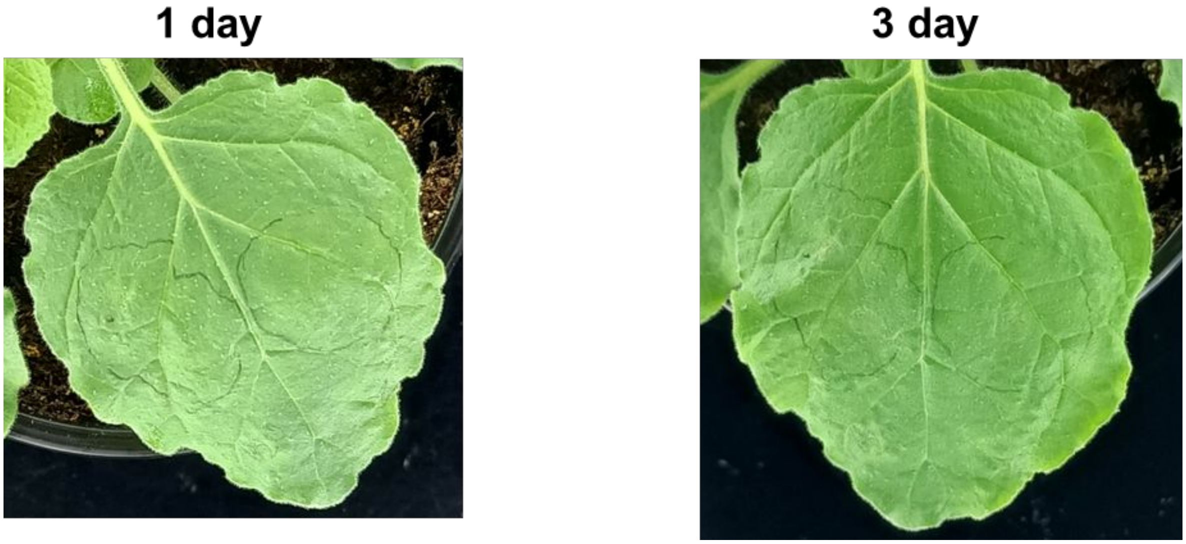

Figure S13. The tissue influence caused by infiltration. Pure DNA and DNA@ZIF-8 NPs solution treated leaf after 1 and 3 days.

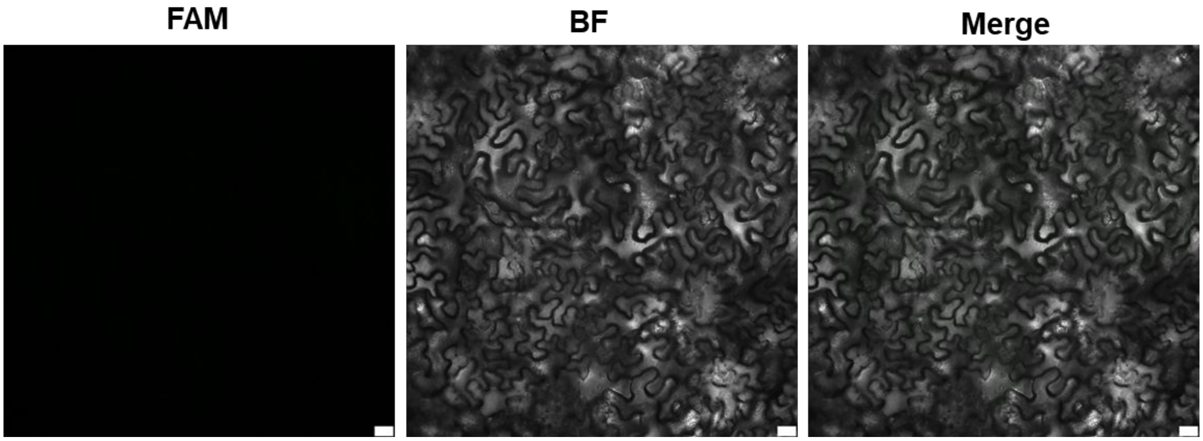

FigureS14. Representative CLSM images of no-treatment area in the same leaf, Scale bar: 20 μm.

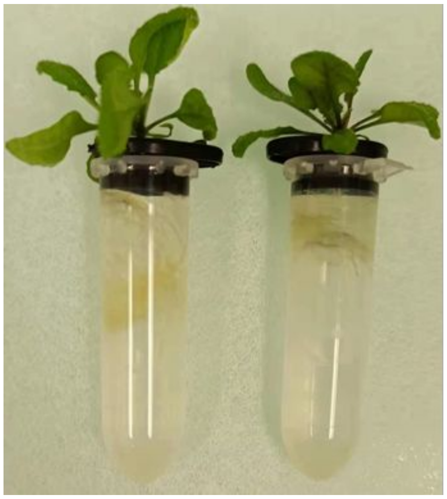

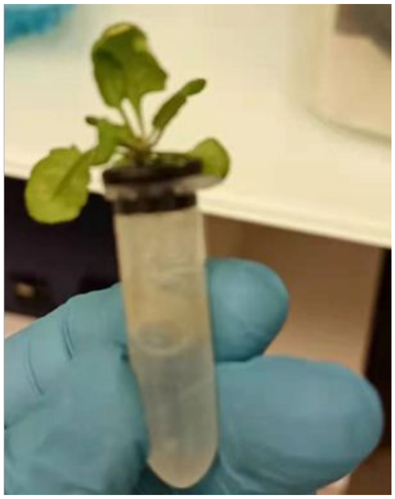

Figure S15. Photographs of *Arabidopsis thaliana* root immersed into mixed pure FAM-DNA or FAM-DNA@ZIF-8 NPs solution.

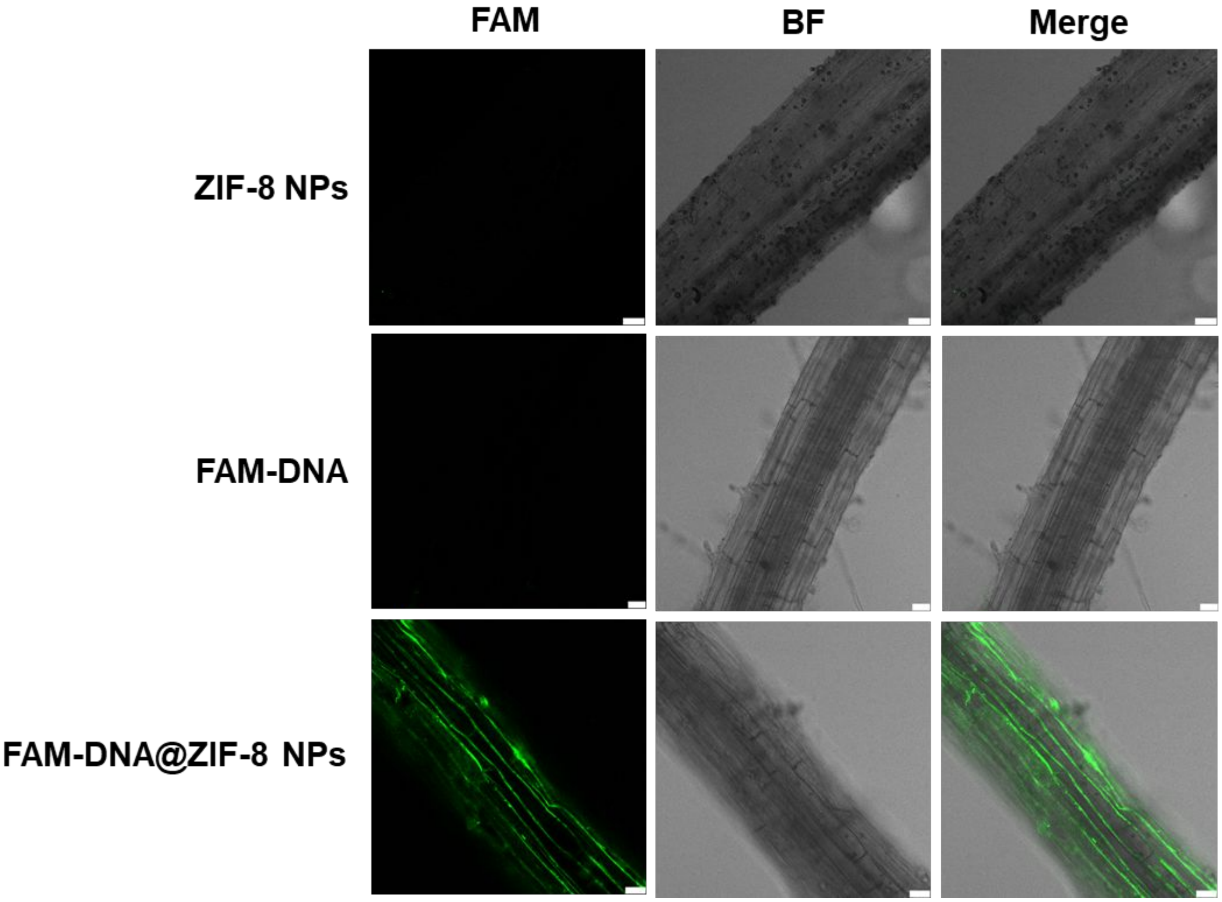

Figure S16. Representative CLSM images of *Arabidopsis thaliana* root cells treated with ZIF-8 NPs, pure FAM-DNA and FAM-DNA@ZIF-8 NPs.

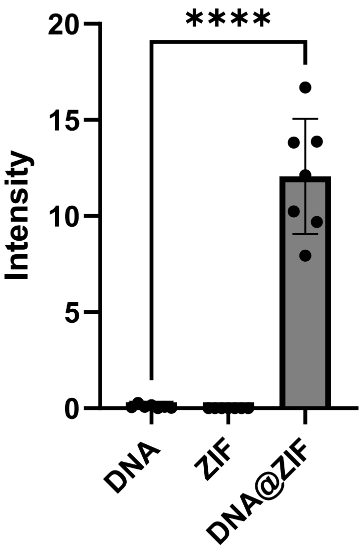

Figure S17. Quantitative analysis of fluorescence intensity in confocal images was conducted for DNA, DNA@ZIF-8 NPs, and ZIF-8 NPs infiltrated into *Arabidopsis thaliana* root cells.****P < 0.0001, SD (n = 7).
